# Cochlear Implantation with a Combined Approach: A Case Report

**DOI:** 10.22038/IJORL.2023.75088.3520

**Published:** 2024-01

**Authors:** Reza Jahangiri, Seyed Basir Hashemi, Elahe Kohan, Amirhossein Babaei

**Affiliations:** 1 *Otolaryngology Research Center, Department of Otolaryngology, Shiraz University of Medical Sciences, Shiraz, Iran.*

**Keywords:** Cochlear implants, Otitis media, Sensorineural hearing loss, Cochlear implantation, Otitis media with effusion

## Abstract

**Introduction:**

Ear symptoms of granulomatosis with polyangiitis can range from ear fullness and otalgia to conductive or sensory neural hearing loss and sudden deafness. Cochlear implantation in these patients faces two challenges: access to the round window and control of mastoid and middle ear inflammation. The combined approach in cochlear implantation is a classic trans-facial recess approach facilitated by a trans-canal view.

**Case Report:**

In this case report, we present the "combined approach" in a 20-year-old lady with granulomatosis with polyangiitis who underwent cochlear implantation successfully using the combined approach.

**Conclusion:**

Post-operative results suggest that the “combine approach” seems to be a safe, easy, and fast cochlear implantation technique for chronic otitis media with an atelectatic middle ear and retracted tympanic membrane or narrow facial recess space. It is a single-stage surgery that has no need for the obliteration of the ear and has less morbidity.

## Introduction

Granulomatosis with polyangiitis (Wegener's granulomatosis) is an uncommon, multi-systemic disease with nasal, laryngeal, and otological manifestations. Symptoms of ear involvement can range from ear fullness and otalgia to conductive or sensory neural hearing loss and sudden deafness. These symptoms can appear before the rheumatologist's definite diagnosis of the disease. Patients with severe or profound hearing loss who did not gain from a hearing aid should be candidates for cochlear implantation (1,2).

Cochlear implantation is usually performed via a trans-facial recess approach. However, some authors suggested alternative techniques, such as the Veria technique and the suprameatal approach, especially for cases that had restricted mastoid cavity or facial nerve anomalies, to reduce surgical complications (3, 4). Cochlear implantation in granulomatosis with polyangiitis patients faces two challenges: access to the round window and controlling mastoid and middle ear inflammation such as tympanic membrane perforation before or after surgery (5). The combined approach is a classic trans-facial recess approach facilitated by a trans-canal view (6).

In this case report, we restored the hearing sense of a patient with granulomatosis with polyangiitis via a “combined approach” cochlear implantation.

## Case Report

**Fig 1:**
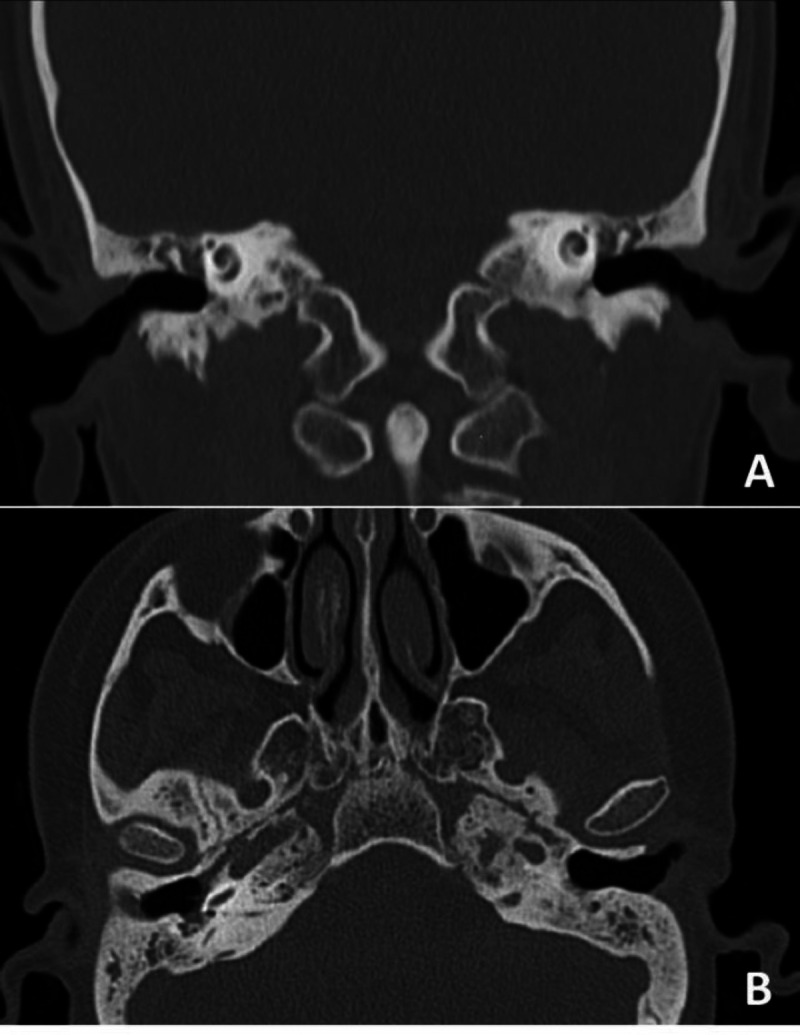
Pre-operative temporal CT scan. A) coronal view, B) axial view

A 20-year-old lady presented first with bilateral otalgia and hearing loss three years ago. Due to a flat tympanogram and conductive hearing loss, serous otitis media was considered for her, and a ventilation tube was inserted bilaterally. Post-operatively, otorrhea, followed by dyspnea and jaw locking, were added to her symptoms. A cystic lesion was detected in the lung computer tomography (CT). According to a positive C-ANCA and a high erythrocyte sedimentation rate (ESR), granulomatosis with polyangiitis was diagnosed. Azathioprine and prednisolone were prescribed to control the disease and a hearing aid for amplifying sounds. Hearing deterioration developed rapidly over two months, causing bilateral deafness. The tympanic membrane was atrophic and retracted with no perforation in physical examination. Tympanometry was B-type bilaterally. A temporal bone CT scan revealed underdeveloped mastoid air cells, the middle ear, and mastoid opacification and retraction of the tympanic membrane (Figures 1A and 1B).

Because of the ineffectiveness of the hearing aid, the patient was decided to be a candidate for right ear cochlear implantation with the nucleus slim modiolar electrode (CI532, Cochlear Ltd., Sidney, Australia). With the postauricular approach, after mastoidectomy and clearing the attic tissue, the short process of the incus became visible. A posterior tympanotomy was performed with difficulty because of bleeding from the inflamed tissue. Thus, the opening of the facial recess was narrow, and the visibility of middle ear components and round window and retraction of the ear drum through the facial recess were insufficient.

After elevation of the tympanomeatal flap, posterior canaloplasty and atticotomy were done. The retracted tympanic membrane was lifted from the middle ear floor and cochlear promontory. Next, the round window was exposed completely. The round window niche was removed with a 0.8mm diamond drill, exposing its membrane. The electrode entered the middle ear through the facial recess and was guided to the round window with the ear canal microscopic view. Therefore, it was fully placed in the scala tympani with the Contour Electrode (Claw Z33021) through the canal.

We used a piece of concha cartilage to reinforce the tympanic membrane to prevent further retraction, perforation of the tympanic membrane, or chronic middle ear infection. Finally, the tympanomeatal flap was returned to its original position. Intraoperative impedance and neural response telemetry (NRT) and post-operative trans-orbital X-ray confirmed the perfect performance and location of the electrode. Post-operative free field audiometry revealed a 30 dB speech reception threshold and an 80% speech discrimination score. After one year, the status of the middle ear was good, and the tympanic membrane was intact.

## Discussion

Ear involvement and otologic manifestations can be seen as initial symptoms or in the continuation of the clinical course of granulomatosis with polyangiitis in 20-60% of patients (7, 8). Conductive hearing loss is the most common form of hearing loss seen in these patients due to dysfunction of the eustachian tube, which can be treated with systemic steroids and ventilation tube placement. However, progressive sensorineural hearing loss is common in these patients (9). 

Sensorineural hearing loss is a sign of disease severity and needs aggressive interventions such as cyclophosphamide prescriptions (10). The definite pathology of sensory neural hearing loss is unknown, although immunocomplex deposition in the inner ear, cochlear nerve involvement by granuloma, and vasculitis of cochlear vessels have been suggested (2).

Although recently Lang et al. reported improvement in SNHL and cochlear signal reduction following immunosuppressive therapy (11), cochlear implantation is still the gold standard method for rehabilitating hearing in the deaf or severe hearing loss when a hearing aid is ineffective (12,13). Cochlear implantation in this disease faces several challenges (1,3,4,5). 

The first challenge is intraoperative bleeding and limited surgical visibility due to inflammatory tissues. The second challenge is the possibility of chronic middle ear infection after implantation. The third challenge is the possibility of intracranial infection and post-operative meningitis. The last challenge is insufficient landmarks and narrow facial recesses which make the facial nerve susceptible to damage.

The literature described different techniques for cochlear implantation in patients with chronic otitis media. Subtotal petrosectomy is an acceptable technique for eradicating ear disease, as described by Fisch and Mattox in 1988. In this method, all air spaces in the temporal bone were eradicated, and the middle ear mucous, tympanic membrane, and external auditory canal skin were removed with the obliteration of the Eustachian tube and external ear canal (14). This method is a very time-consuming operation that sometimes requires two-stage surgery. Also, the possibility of cholesteatoma after surgery is a possible complication. Some surgeons use other methods, such as the Veria method, the modified trans-canal technique (Bhopal technique), and the pericanal electrode insertion technique (3,4,15).

The literature review found only four case reports and one original article about cochlear implantation in a deaf patient following granulomatosis with polyangiitis. The first case was reported in 1996 by Abou-Elhmd et al. using the classic posterior tympanotomy method. Cochlear implantation was done easily, probably owing to acceptable mastoid and middle ear pneumatization, which was not mentioned in their report (2). The second case was reported in 2017 by Elmas and colleagues with subtotal petrosectomy and abdominal fat obliteration (5). 

The third case was reported by Szymanski et al. as the second stage in a patient with an exposed facial nerve and previous subtotal petrosectomy (16). In the last report, a 71-year-old female underwent cochlear implantation without complications (17).

Our patient presented with bilateral serous otitis media and rapidly deteriorating sensorineural hearing loss within two months. It is rare for sensorineural hearing loss to be the initial symptom of granulomatosis with polyangiitis. The patient was referred to our department due to serous otitis media and hearing loss. Initially, we performed ventilation tube insertion as the first-line conservative therapy. Following confirmation of granulomatosis with polyangiitis and the development of profound sensorineural hearing loss, and with no response to medical therapy and limited benefits from hearing aids, we proceeded with cochlear implantation during remission of the underlying disease, resulting in a satisfactory outcome.

## Conclusion

The “Combined approach” seems to be a safe, easy, and fast technique for cochlear implantation in the case of chronic otitis media with an atelectatic middle ear and retracted tympanic membrane or narrow facial recess space. It is a single-stage surgery that has no need for the obliteration of the ear and has less morbidity. 

Also, the otologists seem familiar with this approach and do not need additional expertise. Furthermore, if revision surgery is required, it is easier and does not distract the middle ear structure.
